# Supplementary material for: Optimized intramuscular immunization with VSV-vectored spike protein triggers a superior immune response to SARS-CoV-2
Source: NPJ Vaccines. 2022 Jul 25;7:82. doi: 10.1038/s41541-022-00508-7 (PMC9309237; doi:10.1038/s41541-022-00508-7)
Supplement: Supplementary file 1 — Supplementary Information [file 41541_2022_508_MOESM1_ESM.pdf]

Supplementary Table 1. Histopathologic assessment of lung pathology

Evaluation criteria	Score[1]				
	0	1	2	3	4
Distribution of lesions	Absent	Focal	Multifocal (to coalescing)	Diffuse	
Affected tissue (%)	<1%	2-10%	11-25%	26-50%	>50%
Expansion to peripheral lung regions	No	Yes			
Pulmonary atelectasis	Absent	Minor	Major		
Peribronchial inflammation	Absent	Minor	Major		
Perivascular inflammation	Absent	Minor	Major		
Interstitial inflammation	Absent	Minor	Major		
Intra-alveolar inflammation	Absent	Minor	Major		
Bronchial epithelial necrosis	Absent	Minor	Major		
Alveolar wall necrosis	Absent	Minor	Major		
Infiltration by neutrophils	Absent	Minor	Major		
Infiltration by macrophages	Absent	Minor	Major		
Infiltration by lymphocytes	Absent	Minor	Major		
Alveolar edema	Absent	Minor	Major		
Perivascular edema	Absent	Minor	Major		
Fibrinoid degeneration of vascular walls	Absent	Minor	Major		
Vascular thrombosis	Absent	Minor	Major		
Vasculitis	Absent	Minor	Major		
Hemorrhage (interstitial, intraalveolar)	Absent	Minor	Major		
Pleuritis	Absent	Minor	Major		

[1] The scoring of lung lesions was performed according to Dietert et al, 2017.[51]

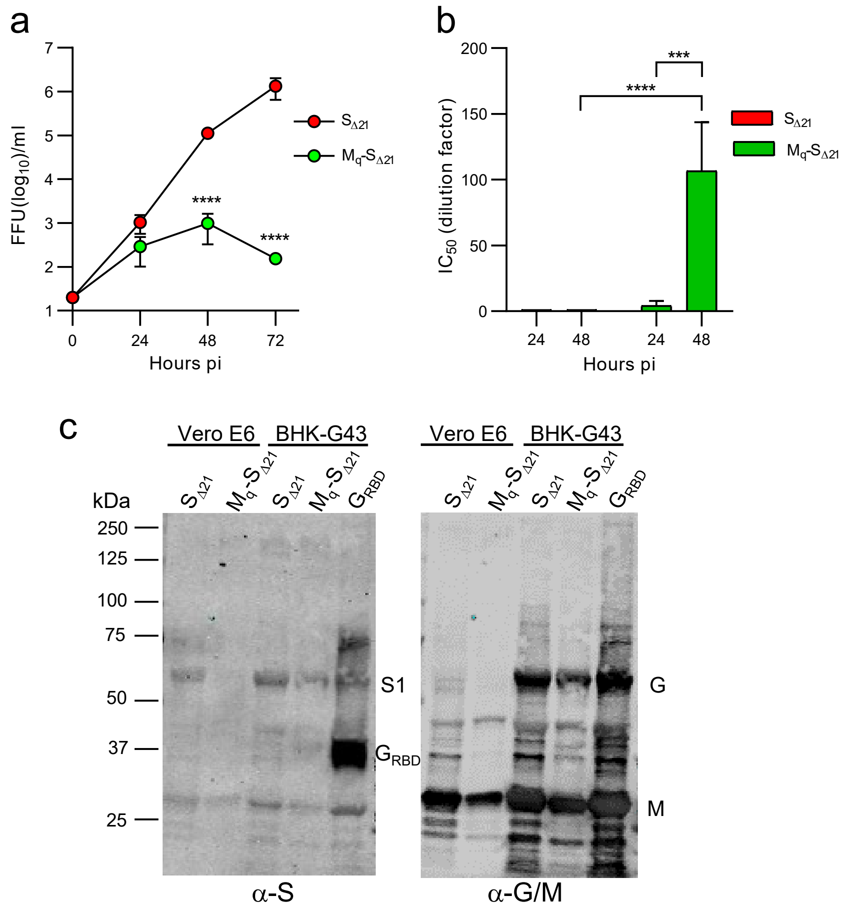

Supplementary Fig. 1. Attenuation of VSV*M_qΔG-$S_{Δ21}$ by the induction of type I interferon. A549-hACE2/hTMPRSS2 cells were infected with either VSV*ΔG-$S_{Δ21}$ or VSV*M_qΔG-$S_{Δ21}$ using an moi of 0.01 ffu/cell. (**a**) Infectious virus produced at the indicated time points was determined by titration of the cell culture supernatants on Vero E6 cells. (**b**) Quantification of antiviral activity in conditioned medium on the VSV*ΔG(FLuc) reporter virus. Mean values and SD were calculated from 3 infection experiments. Statistically different values were computed using the two-way ANOVA test (***p = 0.0005; ****p < 0.0001). (**c**) Analysis of recombinant VSV-vectored vaccines by Western blot after SDS-PAGE under reducing conditions. See also the legend to **Fig. 1** for details.

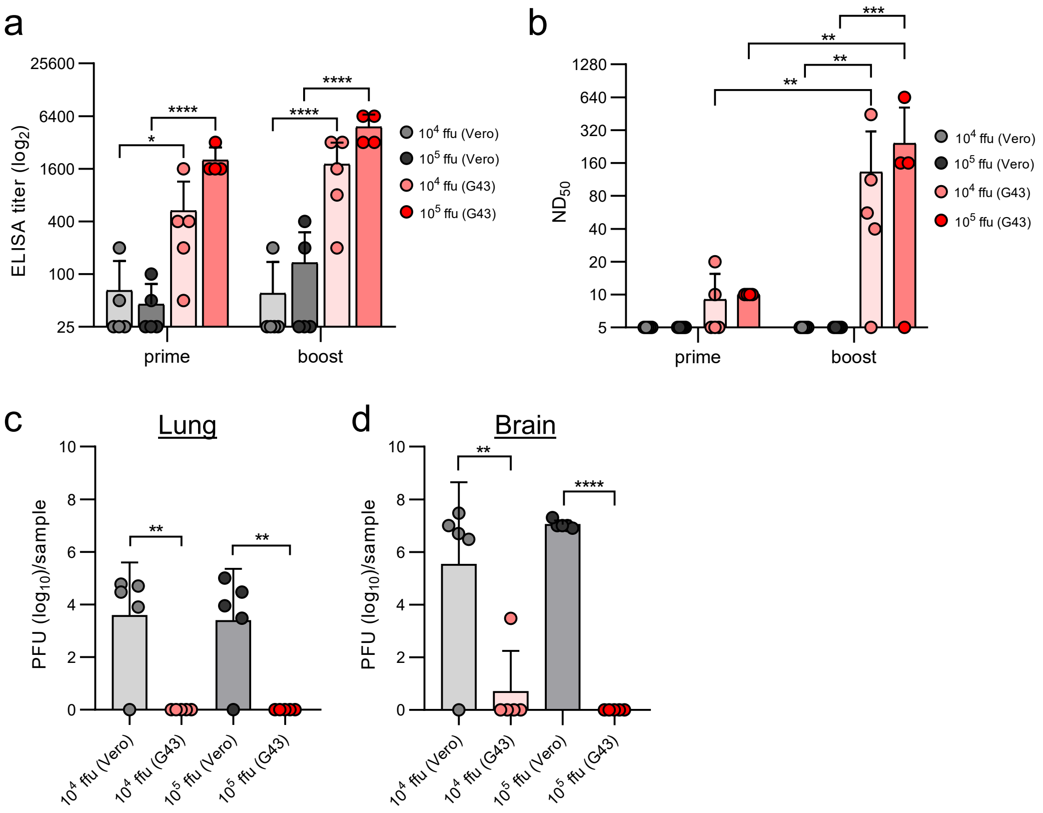

Supplemental Fig. 2. **Comparison of ACE2-dependent and -independent immunization of C57BL/6 mice.** C57BL/6 mice (group size n = 5) were immunized (i.m.) with either 10^4 or 10^5 focus-forming units (ffu) of VSV*ΔG-$S_{\Delta 21}$ vector produced on either Vero E6 or BHK-G43 cells. (**a**) Detection of spike-specific serum antibodies by ELISA 3 weeks after the first (prime) and 3 weeks after the second immunization (boost). (**b**) Determination of the virus neutralization dose 50% (ND_{50}) in serum of immunized mice. (**c, d**) Determination of infectious SARS-CoV-2-S^{D614G} titers in homogenates of lung (**c**) and brain homogenates (**d**) at 9 days pi. Mean values and SD are shown in all data sets. Significantly different values were calculated by the two-way ANOVA test (**b**) and by the one-way ANOVA test (**c, d**) (*$p < 0.05$, **$p < 0.01$; ***$p < 0.0005$, ****$p < 0.0001$).

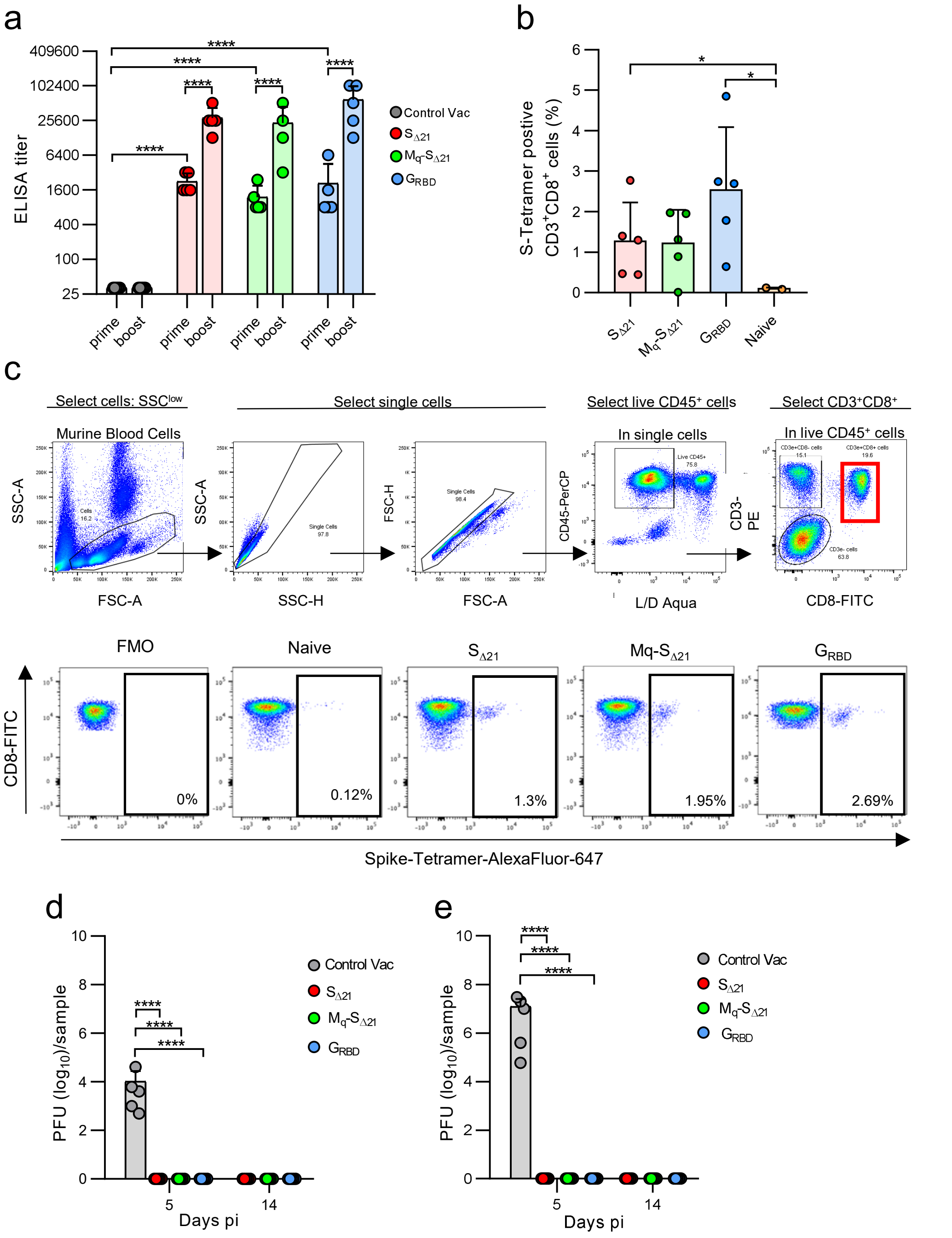

Supplemental Fig. 3. Humoral and cellular immune responses of vaccinated K18-hACE2 mice. (**a**) Detection of spike-specific serum antibodies by ELISA 3 weeks after the first (prime) and 3 weeks after the second immunization (boost) in the indicated vaccine groups (n = 5). (**b**) Frequency of spike protein-specific cells in the $CD3^+/CD8^+$ blood lymphocyte population 3 weeks after the second immunization with the indicated vaccines. (**c**) Representative flow cytometric analysis of $CD3^+/CD8^+$ lymphocytes after staining with the spike-specific tetramers. Upper panel: Gating strategy. Lower panel: Representative tetramer staining of lymphocytes from a single animal per each vaccine group (lower panel). (**d, e**) Determination of infectious SARS-CoV-2 titers by plaque assay in lung (**d**) and brain (**e**) tissue homogenates prepared from animals euthanized at either day 5 or day 14 pi. Mean values (represented by bar heights) and SD are shown. Statistically significant values were determined by the two-way ANOVA test (**a**), the one-tailed Mann-Whitney test (**b**), and the one-way ANOVA test (**d, e**) (*$p < 0.05$, ****$p < 0.0001$).

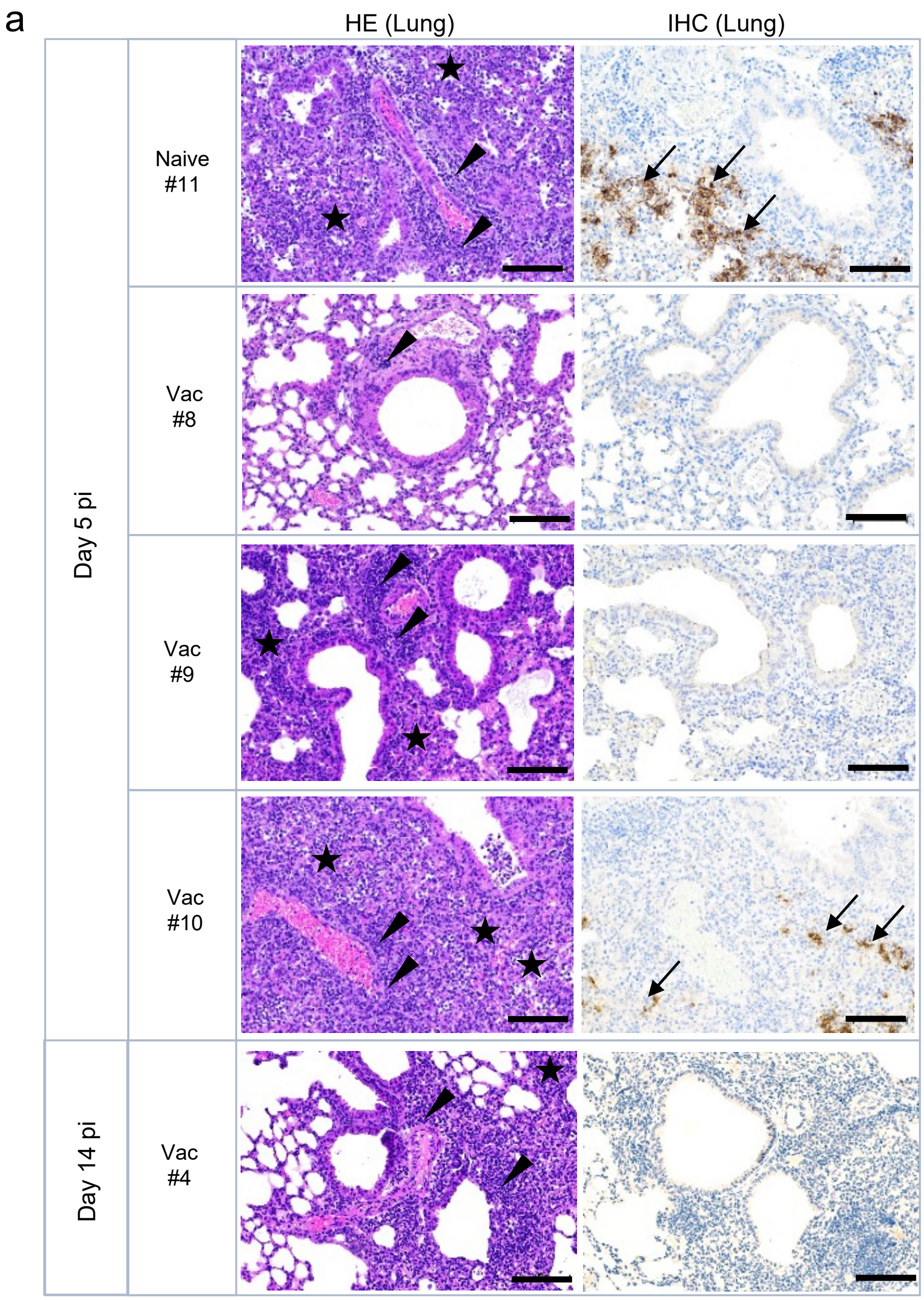

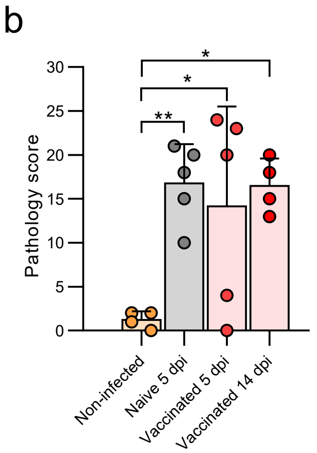

Supplementary Fig. 4. **Histological analysis of lung tissue sections following SARS-CoV-2 Delta VOC infection**. (**a**) Naïve and vaccinated K18-hACE2 mice were euthanized at day 5 and day 14 pi with SARS-CoV-2^{Delta} VOC and lung sections from individual mice analyzed by HE staining (HE Lung) and viral nucleoprotein immunostaining (IHC Lung). Arrow heads indicate perivascular/peribronchiolar infiltration. Stars mark tissue consolidation. Arrows indicate areas where SARS-CoV-2 nucleoprotein antigen was detected by IHC. The animal ID numbers shown on the left side of the figure identify the animals labeled in **Fig. 7c** and **Fig. 7g**. Bars = 100 µm. (**b**) Histopathological scoring of lung tissue sections prepared from vaccinated and naïve mice 5 and 14 days pi. Mean values and SD are indicated. Statistical differences were calculated with the one-way ANOVA test (*$p < 0.05$; **$p < 0.01$).

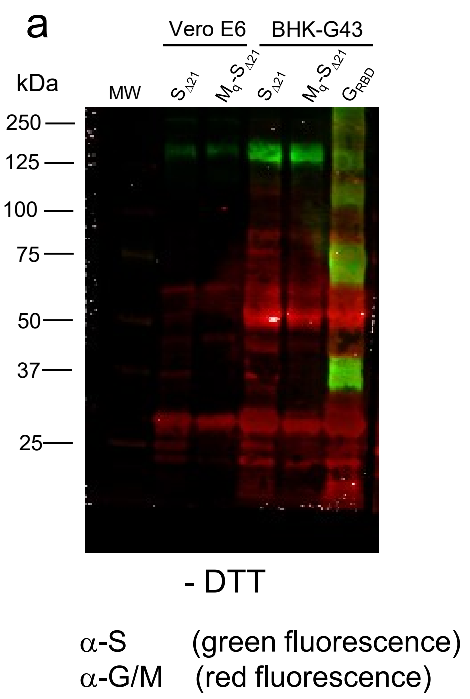

Supplementary Figure 5: **Western blot analysis of recombinant VSV vector particles**. VSV*ΔG-$S_{\Delta21}$ ($S_{\Delta21}$) and VSV*M_qΔG-$S_{\Delta21}$ (M_q-$S_{\Delta21}$) were propagated on both Vero E6 and BHK-G43 cells, while VSV*ΔG-G_{RBD} (G_{RBD}) were propagated only on BHK-G43 helper cells. At 24 hours pi, the virus particles were concentrated from the cell culture supernatant by ultracentrifugation and dissolved in SDS sample buffer. The viral proteins were separated by SDS-PAGE under (**a**) non-reducing (-DTT) or (**b**) reducing (+ DTT) conditions and blotted onto nitrocellulose membrane. Antigens were detected with a COVID-19 convalescent serum (α-S) and a rabbit polyclonal immune serum directed to the VSV G and M proteins (α-G/M). The human convalescent serum was detected with a goat anti-human IRDye 800CW serum (green fluorescence), while the rabbit immune serum was detected with a goat anti-rabbit IRDeye 680RD serum (red fluorescence). The blots were derived from the same experiment and were processed in parallel.
