# Supplementary material for: Optimized intramuscular immunization with VSV-vectored spike protein triggers a superior immune response to SARS-CoV-2
Source: NPJ Vaccines. 2022 Jul 25;7:82. doi: 10.1038/s41541-022-00508-7 (PMC9309237; doi:10.1038/s41541-022-00508-7)
Supplement: Supplementary file 2 — REPORTING SUMMARY [file 41541_2022_508_MOESM2_ESM.pdf]

nature portfolio

Corresponding author(s): Gert Zimmer
Charaf Benarafa

Last updated by author(s): Mar 28, 2022

Reporting Summary

Nature Portfolio wishes to improve the reproducibility of the work that we publish. This form provides structure for consistency and transparency in reporting. For further information on Nature Portfolio policies, see our Editorial Policies and the Editorial Policy Checklist.

Statistics

For all statistical analyses, confirm that the following items are present in the figure legend, table legend, main text, or Methods section.

n/a	Confirmed	
☐	☒	The exact sample size (n) for each experimental group/condition, given as a discrete number and unit of measurement
☐	☒	A statement on whether measurements were taken from distinct samples or whether the same sample was measured repeatedly
☐	☒	The statistical test(s) used AND whether they are one- or two-sided<br>*Only common tests should be described solely by name; describe more complex techniques in the Methods section.*
☐	☒	A description of all covariates tested
☐	☒	A description of any assumptions or corrections, such as tests of normality and adjustment for multiple comparisons
☐	☒	A full description of the statistical parameters including central tendency (e.g. means) or other basic estimates (e.g. regression coefficient) AND variation (e.g. standard deviation) or associated estimates of uncertainty (e.g. confidence intervals)
☐	☒	For null hypothesis testing, the test statistic (e.g. F, t, r) with confidence intervals, effect sizes, degrees of freedom and P value noted<br>*Give P values as exact values whenever suitable.*
☒	☐	For Bayesian analysis, information on the choice of priors and Markov chain Monte Carlo settings
☒	☐	For hierarchical and complex designs, identification of the appropriate level for tests and full reporting of outcomes
☒	☐	Estimates of effect sizes (e.g. Cohen's d, Pearson's r), indicating how they were calculated

Our web collection on statistics for biologists contains articles on many of the points above.

Software and code

Policy information about availability of computer code

Data collection	NBD FACS DIVA v6 for flow cytometry acquisition.
Data analysis	GraphPad Prism program package version 8; FlowJo v10.

For manuscripts utilizing custom algorithms or software that are central to the research but not yet described in published literature, software must be made available to editors and reviewers. We strongly encourage code deposition in a community repository (e.g. GitHub). See the Nature Portfolio guidelines for submitting code & software for further information.

Data

Policy information about availability of data

All manuscripts must include a data availability statement. This statement should provide the following information, where applicable:

- Accession codes, unique identifiers, or web links for publicly available datasets
- A description of any restrictions on data availability
- For clinical datasets or third party data, please ensure that the statement adheres to our policy

The datasets generated during and/or analysed during the current study are available from the corresponding author on reasonable request.

Field-specific reporting

Please select the one below that is the best fit for your research. If you are not sure, read the appropriate sections before making your selection.

☒ Life sciences ☐ Behavioural & social sciences ☐ Ecological, evolutionary & environmental sciences

For a reference copy of the document with all sections, see nature.com/documents/nr-reporting-summary-flat.pdf

Life sciences study design

All studies must disclose on these points even when the disclosure is negative.

Sample size	A power analysis using the online software of Columbia University Medical Center (www.biomath.info) was performed to calculate the group size for animal experiments.
Data exclusions	No data were excluded from the analyses.
Replication	Mice immunization studies with trans-complemenetd and non-transcomplemented vector vaccine was performed in groups of 5 mice and was replicated with two different vaccine doses and in two different mouse models. Challenge infection studies (with measurements of body weight, virus load in oropharyngeal swabs and organs and infectious virus titers in lungs and brains), were performed with groups of 5 mice and were replicated with groups euthanized at day 5 and day 14 post infection. Virus-neutralization and pseudotype virus neutralization tests were operformed with sera from 10 correspondingly immunized mice. Determination of frequency of spike-specific CD8+ cells by tetramer staining was done with groups of 5 mice. All attempts at replication were sucessful.
Randomization	Mice were randomly allocated to experimental groups.
Blinding	Histopathology analysis was performed in a blinded manner. All other experiments were performed not blinded.

Reporting for specific materials, systems and methods

We require information from authors about some types of materials, experimental systems and methods used in many studies. Here, indicate whether each material, system or method listed is relevant to your study. If you are not sure if a list item applies to your research, read the appropriate section before selecting a response.

Materials & experimental systems

n/a	Involved in the study	
☐	☒	Antibodies
☐	☒	Eukaryotic cell lines
☒	☐	Palaeontology and archaeology
☐	☒	Animals and other organisms
☒	☐	Human research participants
☒	☐	Clinical data
☒	☐	Dual use research of concern

Methods

n/a	Involved in the study	
☒	☐	ChIP-seq
☐	☒	Flow cytometry
☒	☐	MRI-based neuroimaging

Antibodies

Antibodies used	Rabbit polyclonal anti-VSV-G/M immune serum (Georg Herrler, University of Veterinary Medicine, Hannover, Germany); rabbit polyclonal anti-SARS-CoV nucleocapsid serum (Rockland, 200-401-A50); human convalescent COVID-19 patient serum (Nina Khanna, University of Basel, Switzerland); PerCP/Cyanine5.5 anti-mouse CD45 antibody, clone 30-F11 (Biolegend, Cat No. 103132); PE anti-mouse CD3epsilon antibody, clone 145-2C11 (Biolegend, Cat. No. 100308), FITC anti-mouse CD8a antibody, clone 53-6.7 (Biolegend, Cat No. 100706); goat anti-mouse immunoglobulins HRP conjugate (Agilent-Dako, Cat No. P0260); IRDye 800CW goat anti-human IgG (Li-Cor Biosciences, Cat No. 926-322232); IRDye 680RD goat anti-rabbit IgG (Li-Cor Biosciences, Cat No. 926-68071);
Validation	The rabbit polyclonal anti-VSV-G/M immune serum has been validated by Western blot (Hoffmann et al. 2010, J. Gen. Virol. 91, 2782-2793). The convalescent COVID-19 patient serum was evaluated for virus-neutralizing activity (Sava et al. 2021 Swiss Med Wkly 151:w20550) and for binding to the SARS-CoV-2 spike protein by Western blot (present study). Detailed informations on the commercially available primary antibodies used are available on the web pages of Biolegend (www.biolegend.com) and Rockland (www.rockland.com).

Eukaryotic cell lines

Policy information about cell lines

Cell line source(s)	Vero E6 (Christian Drosten, Charite, Berlin, Germany); Vero-TMPRSS2 (Markus Hoffmann, German Primate Center, Göttingen, Germany); A549-hACE2-hTMPRSS2 (Invivogen, Tolouse, France); A549 (German Cell Culture collection, DSMZ; Braunschweig, Germany); BHK-21 (American Type Culture Collection ATCC, CCL-10); I1 Hybridoma cells (ATCC, CRL-2700); BHK-G43 (Gert Zimmer, IVI, Bern & Mittelhäusern, Switzerland)
Authentication	None of the cell lines used were authenticated.
Mycoplasma contamination	All cell lines were tested negative for contamination by mycoplasms.
Commonly misidentified lines (See ICLAC register)	Commonly misidentified cell line were not used in this study.

Animals and other organisms

Policy information about studies involving animals; ARRIVE guidelines recommended for reporting animal research

Laboratory animals	C57BL/6 mice (male, 12-18 weeks old); Transgenic Tg(K18-hACE2)2Prlmn mice (female, 17-41 weeks old)
Wild animals	The study did not involve wild animals.
Field-collected samples	The study did not involve samples collected from the field.
Ethics oversight	Animal experiments have been approved by the animal welfare committee of the Canton of Bern (authorization number: BE43/20).

Note that full information on the approval of the study protocol must also be provided in the manuscript.

Flow Cytometry

Plots

Confirm that:

☒ The axis labels state the marker and fluorochrome used (e.g. CD4-FITC).

☒ The axis scales are clearly visible. Include numbers along axes only for bottom left plot of group (a 'group' is an analysis of identical markers).

☒ All plots are contour plots with outliers or pseudocolor plots.

☒ A numerical value for number of cells or percentage (with statistics) is provided.

Methodology

Sample preparation	Stain was performed in EDTA-anticoagulated whole blood . Red-cell were lysed using standard ACK (Ammonium-Chloride-Potassium) Lysing Buffer.
Instrument	BD FACSCanto™ II system
Software	BD FACS Diva v6
Cell population abundance	No sorting was performed
Gating strategy	FSc/SSc gating was used to select the lymphocyte. On the lymphocyte population, single cells were selected and then CD45+ viable cells were selected. All these selections were done based on the unstained cells. On this population the frequency of tetramer-positive cells were determined based on fluorescence-minus-one control as well as on unrelevant AlexaFluor-647 loaded tetramer.

☒ Tick this box to confirm that a figure exemplifying the gating strategy is provided in the Supplementary Information.
